# Regulation of Apoptosis, Autophagy, and Metastasis by Luteolin in Human Bladder Cancer EJ138 Cells: An Experimental Study

**DOI:** 10.5812/ijpr-153408

**Published:** 2024-11-16

**Authors:** Mohammad Amin Vatankhah, Alireza Ziyabakhsh, Mohammad Vakili Ojarood

**Affiliations:** 1 Cancer Immunology and Immunotherapy Research Center, Ardabil University of Medical Sciences, Ardabil, Iran; 2 Department of Radiology, Tabriz University of Medical Sciences, Tabriz, Iran; 3 Student Research Committee, School of Medicine, Ardabil University of Medical Sciences, Ardabil, Iran; 4 Department of Surgery, Ardabil University of Medical Sciences, Ardabil, Iran

**Keywords:** Bladder Cancer, Luteolin, Apoptosis, Autophagy, Metastasis

## Abstract

**Background:**

Chemotherapy remains a primary approach to cancer treatment, widely applied in bladder cancer (BC). However, the various side effects and resistance associated with chemotherapeutic drugs pose significant challenges in BC therapy, prompting interest in natural compounds like luteolin. Studies focus on its effects on key biological processes involved in BC, including metastasis, apoptosis, and autophagy.

**Objectives:**

This study investigated the regulation of mRNA expression of genes associated with apoptosis (BCL2, P53), autophagy (ULK1, ATG12), and metastasis (MMP2, MMP9) in malignant BC cells treated with luteolin.

**Methods:**

This was an in vitro experimental study. EJ138 BC cells were treated with various concentrations of luteolin, and its impact on cell viability, proliferation, and gene expression was assessed.

The cytotoxic effect of luteolin on EJ138 BC cells was evaluated using the MTT assay after 24- and 48-hour treatments with different luteolin concentrations. Flow cytometry was performed to examine luteolin’s anti-proliferative effect, and RT-PCR was used to analyze mRNA expression of BCL2, P53, ULK1, ATG12, MMP2, and MMP9 genes.

**Results:**

MTT assay results confirmed that luteolin reduced the proliferation rate of BC cells. Flow cytometry indicated increased cell death in EJ138 BC cells following luteolin treatment. RT-PCR findings demonstrated that luteolin upregulated P53, ULK1, and ATG12 expression while downregulating BCL2 mRNA expression. However, luteolin treatment in EJ138 cells did not significantly alter MMP2 and MMP9 expression levels.

**Conclusions:**

These findings indicate that luteolin exerts cytotoxic effects on EJ138 BC cells by dysregulating mRNA expression of genes involved in apoptosis and autophagy. Therefore, luteolin shows potential as an effective anti-cancer agent for BC therapy.

## 1. Background

Bladder cancer (BC) is the thirteenth leading cause of cancer-related deaths globally, with a 5-year survival rate of 77% (1). In 2018, approximately 550,000 people were diagnosed with BC, accounting for around 3% of all new cancer cases. Greece and Lebanon have the highest incidence rates of BC among men and women (2). Tobacco smoking is the primary risk factor for BC, contributing to roughly 50% of cases. Other significant risk factors include alcohol, cannabis, and opium use, low fiber and grain intake, caffeine, chronic inflammation, arsenic, and nitrates (3). Currently, surgical resection, chemotherapy, radiotherapy, and immunotherapy are the primary treatment methods for BC, playing a crucial role in improving the 5-year survival rate and potential cure of BC patients (4). Despite advancements, various biological pathways and genes involved in key processes such as autophagy, apoptosis, and metastasis continue to influence BC cell proliferation, migration, and poor prognosis in BC patients (5).

Autophagy is a vital cellular process that maintains cellular integrity by recycling metabolic elements under stress conditions and is significantly involved in cancer cell development (6). Recent studies have shown that the dysregulation of autophagy-related genes, such as ATG12 and ULK1, plays a crucial role in the progression of various cancers, including gastric cancer, acute myeloid leukemia, renal cell carcinoma, and BC (7-9). P53 and BCL2 are essential in controlling the proliferation of malignant cancer cells by regulating apoptosis. Studies have indicated that up-regulating P53 and down-regulating BCL2 can suppress the growth of different cancer cells by inducing apoptosis (10). Conversely, down-regulating MMP2 and MMP9 has been shown to reduce metastasis and migration in retinoblastoma, bladder, oral, and ovarian cancer cells (11). Targeting these genes with novel agents could provide new treatment approaches for BC by regulating autophagy, apoptosis, and metastasis.

There is increasing interest in exploring herbal pharmacological agents capable of modulating multiple genes across various tumor types. Among these plant-based agents, luteolin—a flavonoid commonly found in various vegetables, plants, and fruits—has gained attention. Recent research has demonstrated that luteolin plays a significant role in several biological processes, including managing allergy, inflammation, diabetes, and neuroprotection (12). Additionally, these studies have revealed that luteolin exhibits anti-cancer effects by modifying molecular targets, regulating signaling pathways, and altering cellular processes such as apoptosis, cell cycle progression, metastasis, and autophagy, which collectively reduce invasion, migration, and proliferation in cancer cells (13). Luteolin has been shown to increase the expression of pro-apoptotic genes, such as P53 and Bax, while down-regulating anti-apoptotic genes like BCL2 and Bcl-xL (14). Luteolin’s ability to regulate MMP expression has made it a promising compound for preventing or slowing down cancer cell migration from the primary tumor site to distant tissues (15). Additionally, luteolin’s capacity to modulate autophagy is particularly relevant in cancer therapy and inflammation, where autophagy dysfunction plays a crucial role (16).

## 2. Objectives

Luteolin, a plant-based compound, shows potential in the prevention and treatment of various cancers. In this study, we investigate the anti-cancer effects of luteolin on EJ138 BC malignant cells by examining its impact on autophagic, apoptotic, and metastatic gene modulation, specifically targeting ATG12, ULK1, P53, BCL2, MMP2, and MMP9.

## 3. Methods

### 3.1. Study Design

This laboratory-based in vitro study involved exposing EJ138 BC cells to various concentrations of luteolin to assess its effects on cell viability, proliferation, and gene expression.

### 3.2. Chemicals and Reagents

Luteolin and MTT were obtained from Sigma Aldrich. Luteolin was dissolved in RPMI and stored in a refrigerator at 4°C.

### 3.3. Cell Culture

The EJ138 BC cell line was obtained from the National Cell Bank of Iran. This cell line was cultured in RPMI-1640 (Gibco) medium supplemented with 10% heat-inactivated FBS (Gibco), 50 μg/mL streptomycin (Sigma Aldrich), and 50 units/mL penicillin (Sigma Aldrich). The EJ138 BC cells were then maintained in an incubator with 5% CO₂ at 37°C.

### 3.4. Cell Viability Assay

The viability of EJ138 BC cells was assessed using the MTT colorimetric assay. In brief, 1×10^4^ BC cells were seeded into each well of 96-well plates. After a 24-hour incubation, the BC cells were treated with various concentrations of luteolin (10 - 50 µM) for 24 and 48 hours. Subsequently, EJ138 BC cells were washed with PBS, and 20 µL of MTT solution was added to each well. The MTT solution was removed after 4 hours and replaced with 150 µL of DMSO (Scharlau Chemie). Finally, the 570 nm UV absorbance of EJ138 BC cells was measured using an ELISA plate reader.

### 3.5. Flow Cytometric Analysis for Apoptosis Characterization

After the EJ138 BC cells were seeded in a six-well plate (5 × 10^5^ cells/well), 17.92 µM luteolin (corresponding to its IC_50_ value for the EJ138 BC cell line at 48 hours) was applied to treat these cancerous cells. Following 48 hours of treatment, the EJ138 cells were stained with FITC-conjugated annexin V and propidium iodide (PI) for 15 minutes. In the final step, apoptotic EJ138 cells were detected using a flow cytometer (17).

### 3.6. RNA Extraction and Real-time PCR

In the first step, total RNA was extracted from EJ138 BC cells using trizol, following the manufacturer’s instructions (Invitrogen, USA). Three micrograms of total RNA were reverse transcribed using the SMOBIO Kit (RP1300, Taiwan) according to the manufacturer's protocol. Real-time PCR was then performed with 2X qPCRBIO SYGreen Mix Lo-ROX (PCRBiosystems, England). The RT-PCR Detection System (Ruche Light Cycler 96, Germany) was used to analyze gene expression. Oligo primer analysis software (version 7.60) was employed for primer design. GAPDH was used as a housekeeping gene to serve as an internal control for normalization. Relative mRNA expression levels were determined using the ΔCT method (18).

### 3.7. Statistical Analysis

In this study, SPSS V.22 was used to analyze the data. The statistical significance of the differences was assessed using Student’s *t*-test. A P-value of < 0.05 was considered statistically significant.

## 4. Results

### 4.1. Luteolin Induces the Cellular Death in EJ138 Bladder Cancer Malignant Cells

**Figure 1.**
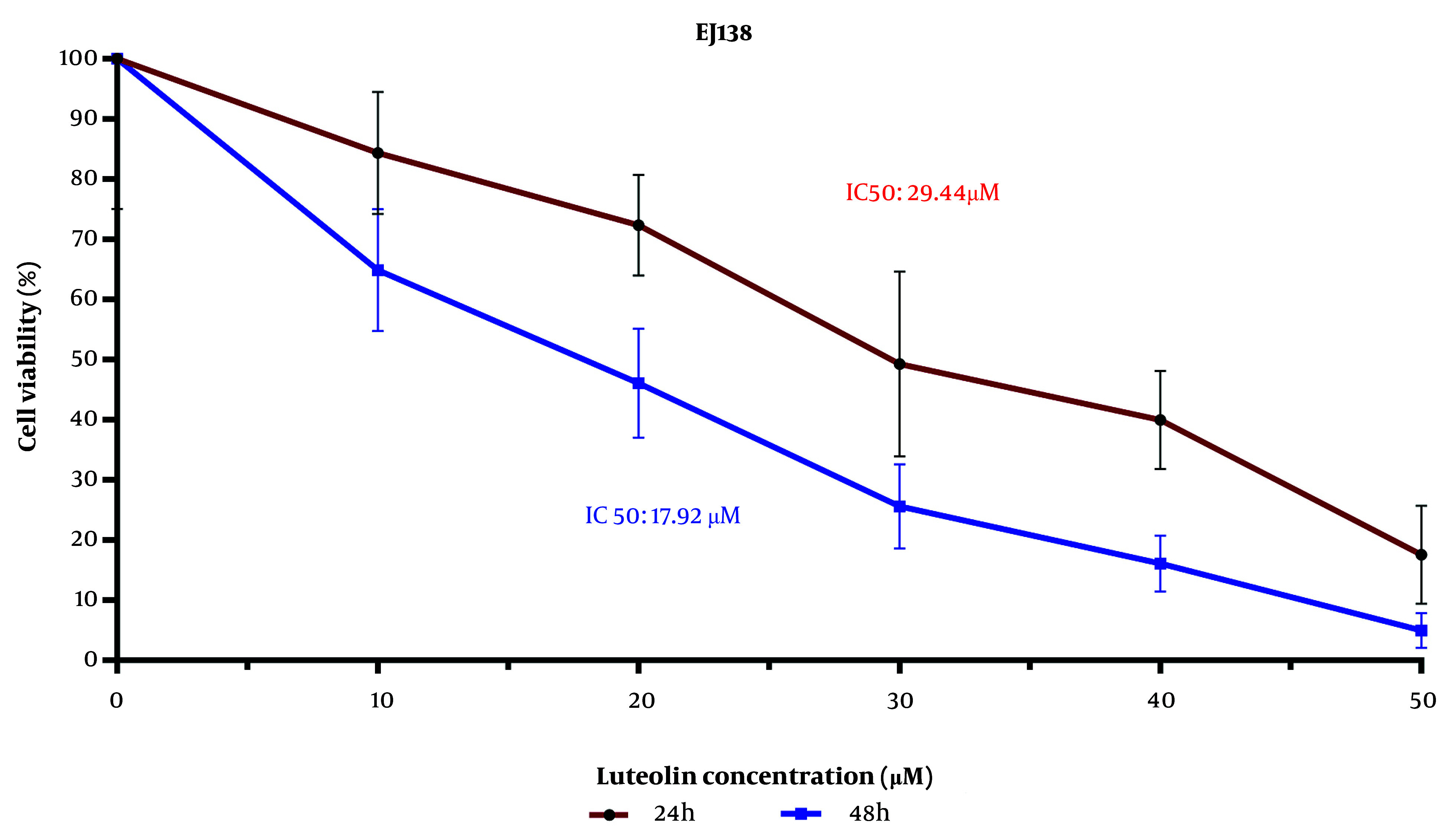
Luteolin treatment raises significant cell death in bladder cancer (BC) cells. Cell viability analysis of EJ138 bladder cancerous cells, utilizing a time- and dose-dependent examination by MTT, was performed for 24 h and 48 h. EJ138 BC cells were treated with 10 - 50 µM luteolin.

The examination of BC cell viability using the MTT assay indicated that the IC_50_ values of luteolin's effect on the EJ138 BC cell line were 29.44 μM and 17.92 μM for 24 h and 48 h, respectively (Figure 1). As shown in Figure 1, cell viability decreased as the luteolin dose increased from 10 μM to 50 μM. These findings illustrate that the growth rate of EJ138 BC cells was inhibited by luteolin in a dose- and time-dependent manner.

### 4.2. Luteolin Enhances the Apoptosis Rate in EJ138 Bladder Cancer Malignant Cells

**Figure 2.**
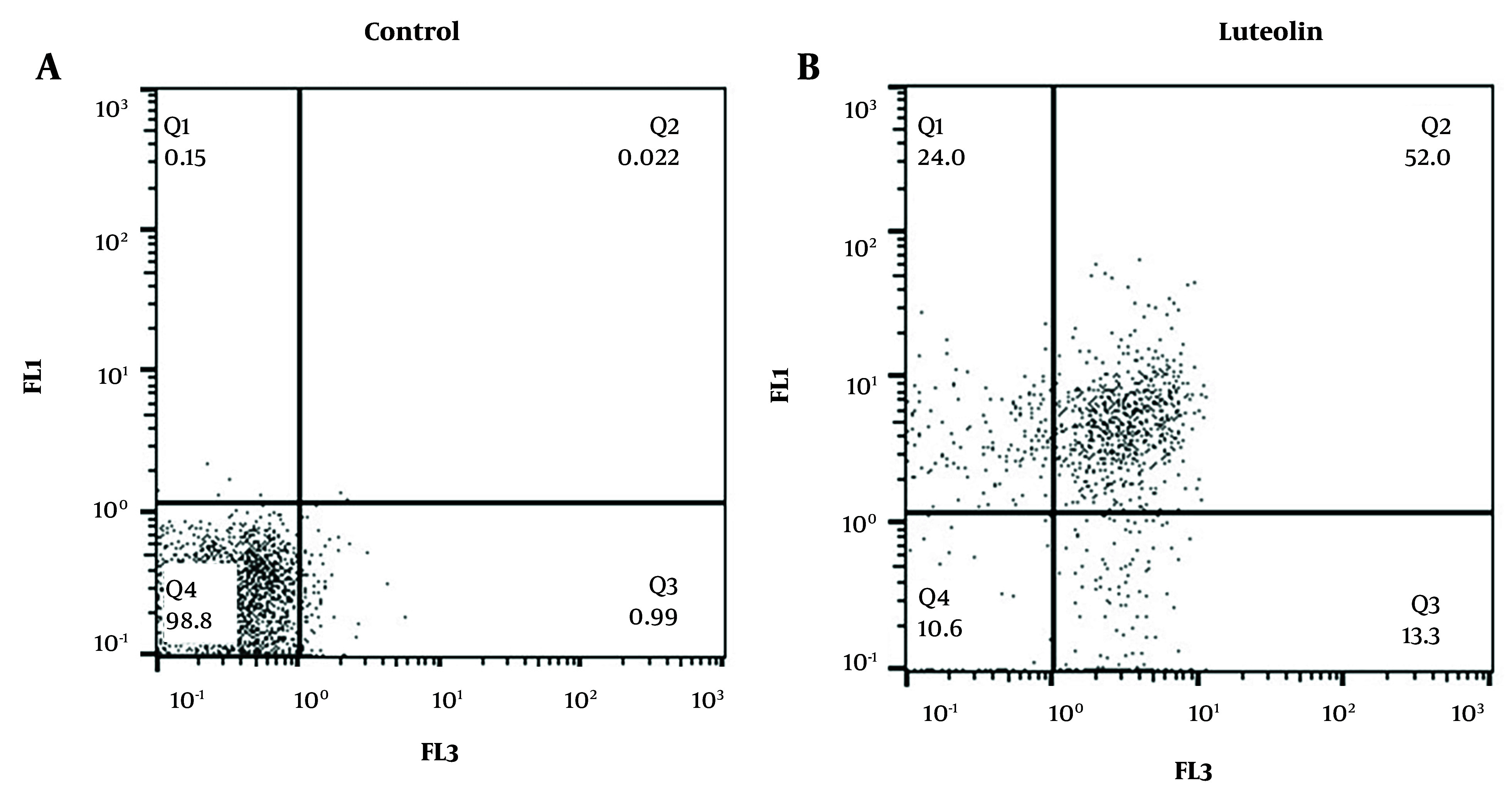
Flow cytometry was conducted to analyze the impacts of luteolin on the apoptosis of EJ138 bladder cancer (BC) cells after 48 h treatment with luteolin. Q1: Necrotic cells, Q2 and Q3: Apoptotic cells, Q4: Live cells.

To investigate whether luteolin mediates its cell death effects through apoptosis, EJ138 BC malignant cells were treated with 17.92 µM luteolin and analyzed by flow cytometry. The results of this investigation showed that treatment with luteolin increased apoptosis in the cancerous cells compared to the control group. As illustrated in Figure 2, luteolin enhanced the apoptotic rate in BC cells by 52%.

### 4.3. Effectiveness of Luteolin in mRNA Expression Levels of Autophagic, Apoptotic, and Metastatic Genes

**Figure 3.**
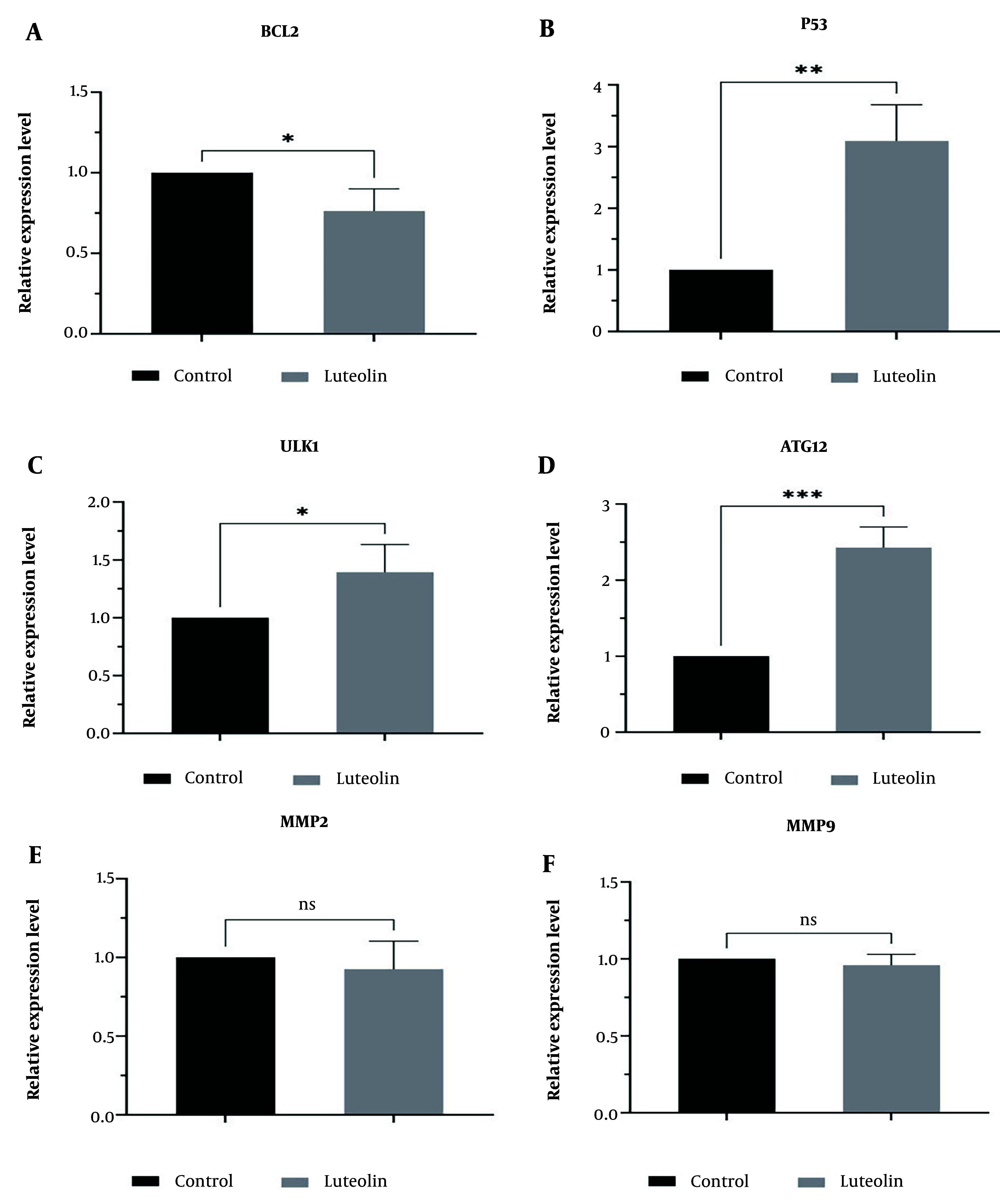
The relative expression of BCL2 (A), P53 (B), ULK1 (C), ATG12 (D), MMP2 (E), and MMP9 (F) was examined by RT-PCR at 48 h and normalized to GAPDH mRNA level post-treatment of EJ138 bladder cancer (BC) cells with luteolin. (* P-value < 0.05, ** P-value < 0.01, and *** P-value < 0.001).

In this study, we used real-time PCR to assess the roles of luteolin in regulating the mRNA expression levels of autophagic, apoptotic, and metastatic genes in EJ138 BC cells. Our findings showed that luteolin upregulated the mRNA expression of the P53, ULK1, and ATG12 genes (Figure 3B, C, D). We also confirmed that luteolin plays a crucial role in inducing apoptosis in EJ138 BC cells by downregulating BCL2 mRNA expression (Figure 3A). In contrast, treatment of BC cells with luteolin did not show a significant difference in the expression levels of MMP2 and MMP9 compared to untreated cells (Figure 3E, F).

## 5. Discussion

Over recent years, significant advancements have been made in treating patients with various malignant cancers due to the development of anti-cancer agents (19). However, many cancer patients continue to suffer due to recurrence, diverse side effects of chemotherapeutic agents, and chemo-resistance (20). For these reasons, numerous studies have been conducted to explore new plant-based anti-cancer agents, such as flavonoid compounds, with fewer adverse effects on cancer patients (21, 22). Although various efforts have been undertaken to investigate the anti-cancer effects of luteolin against cancers, the relationship of metastasis, apoptosis, and autophagy with luteolin has not yet been explored. Therefore, investigating the association between luteolin and biological processes may be an essential step in overcoming various malignant cancerous cells, especially BC (20).

In this present study, we demonstrated that luteolin therapy significantly increased the number of apoptotic BC malignant cells compared with the untreated group. Similar to our study, Wu et al. revealed that luteolin markedly increased the apoptotic rate of triple-negative breast cancer (TNBC) cells. They showed that luteolin decreased the expression of SGT1 and AKT3 genes while increasing BNIP3 mRNA levels, which led to reduced proliferation, invasion, and migration of TNBC (23). In another study, Zhang et al. demonstrated that luteolin promoted cell death and inhibited colony formation in lung cancer cells by downregulating cyclin-D1, P-LIMK, and P-cofilin mRNA expression levels. They also confirmed that luteolin exerted its anti-cancer effects by suppressing P-cofilin, P-LIMK, and Ki-67 expression in lung cancer in vivo (24). Luteolin also promotes apoptosis in SMMC-7721 hepatocellular carcinoma cells by upregulating caspase-8 and downregulating BCL2 protein and mRNA levels (25). In 2020, Lida et al. illustrated that luteolin reduced the viability of T24 BC cells and induced G2/M cell-cycle arrest by increasing p21 and TRX1 and inhibiting p-S6 expression, which plays a crucial role in regulating the mTOR signaling pathway. Their in vivo findings showed that luteolin downregulated p-S6 and the Ki67-Labeling Index in rats, leading to a reduction in BC size (26). Furthermore, combination therapy of luteolin and TRAIL inhibited the growth of T24 BC cells by XIAP suppression and Bax upregulation (27).

Given the extensive apoptotic, autophagic, and anti-metastatic effects of luteolin in various cancer cells, few studies have examined the mechanisms involved (9). Therefore, in this study, we assessed changes in mRNA expression levels of genes that play critical roles in apoptosis, autophagy, and metastasis. The findings of this study illustrated that luteolin exerted its anti-proliferative effects by downregulating BCL2 and upregulating P53. We also showed that luteolin plays an essential role in the autophagy process of BC cells by upregulating ULK1 and ATG12. However, our results did not show significant changes in the expression levels of metastatic genes, including MMP2 and MMP9, in EJ138 BC cells. For this reason, to understand the exact mechanism of luteolin in BC cell metastasis, we suggest using a western blot assay in future studies. 

In 2023, Yajie et al. reported that luteolin enhanced cell death rates and reduced cell proliferation by downregulating BCL2 and P-AKT and upregulating cytochrome C, Bax, and caspase-3 in MKN-45 gastric cancer cells (28). Chang et al. found that luteolin decreased cell growth and sensitized ovarian cancer cells to cisplatin by increasing P53 mRNA expression and decreasing VRK1 expression (29). Contrary to our study, Liu et al. reported that luteolin and ellagic acid reduced ovarian cancer cell metastasis by inhibiting MMP2 and MMP9 protein expression (30). Additionally, luteolin inhibited proliferation, invasion, and metastasis in A375 melanoma cells by downregulating MMP2 and MMP9 and upregulating TIMP-1 and TIMP-2 expression levels (31). Wang et al. indicated that luteolin has critical functions in reducing lung cancer cell metastasis by upregulating miR-106a and downregulating its target genes, including MMP2 and TWIST1 (32). 

Although in vivo evidence suggests that luteolin has potential therapeutic effects, there is a significant gap in clinical trials investigating its efficacy and safety in human BC patients. More comprehensive studies, including long-term animal models and clinical trials, are necessary to determine luteolin’s therapeutic potential and safety in the treatment of human BC.

### 5.1. Conclusions

Altogether, the outcomes of our investigation illustrate that luteolin induces cell death in a dose- and time-dependent manner and inhibits the proliferation of BC cells. Furthermore, our findings demonstrated that luteolin has the potential to be considered as an adjuvant or complementary treatment alongside chemotherapy due to its multi-targeted effects on cancer cells in treating BC patients. Specifically, luteolin upregulates the mRNA expression of P53, ULK1, and ATG12 and downregulates BCL2 expression, genes that play crucial roles in apoptosis and autophagy processes. Therefore, the results of our experimental study provide a scientific basis suggesting that luteolin could offer a promising strategy in BC therapy through its substantial impact on regulating various biological mechanisms. Nonetheless, further investigations are warranted to clarify the exact mechanism of luteolin and explore BC therapy strategies.

## Data Availability

The dataset presented in the study is available on request from the corresponding author during submission or after publication.
